# Low-Voltage Icing Protection Film for Automotive and Aeronautical Industries

**DOI:** 10.3390/nano10071343

**Published:** 2020-07-09

**Authors:** Liberata Guadagno, Fabiana Foglia, Roberto Pantani, Maria Dolores Romero-Sanchez, Blanca Calderón, Luigi Vertuccio

**Affiliations:** 1 Department of Industrial Engineering, University of Salerno, Via Giovanni Paolo II, 132, 84084 Fisciano (SA), Italy; ffoglia@unisa.it (F.F.); rpantani@unisa.it (R.P.); 2 Applynano Solutions S.L., Parque Científico de Alicante, Naves de Apoyo, 3, Zona Ampliación del Campus, 03005 Alicante, Spain; md.romero@applynano.com (M.D.R.-S.); blanca.calderon@applynano.com (B.C.)

**Keywords:** graphene derivatives, smart materials, aspect ratio, electrical heating, de-icing

## Abstract

High-performance heater films are here proposed. They manifest great applicative potentiality in the de-icing technology of aircraft and motor vehicles. The films are suitable to be integrated into composite structures for the de/anti-icing function, which can be activated if the need arises. The heating is based on the joule effect of the current flowing through the electrically conductive films. Voltage and current parameters have been set based on the generators’ capacities on-board an aircraft and a car, as well as on the energy consumption during the operating conditions and the autonomy in the time. Green processes have been employed through all preparative steps of the films, which are composed of expanded graphite (60% wt/wt) and polyvinyl alcohol (PVA) (40% wt/wt). The results reveal a very significant influence of the aspect ratio of the filler on the heating and de-icing performance and suggest how to enhance the de-icing efficiency saving energy and adapting the current on-board aircraft/car generators for de-icing operations.

## 1. Introduction

The icing has relevant effects on the flying safety of an aircraft and the greenhouse gas emissions in the environment. Ice on aircraft surfaces modifies the smooth flow of air, decreasing lift, increasing the mechanical resistance determined by the contact of the aircraft with the air and determining a decrease in the stability. The increase in mechanical resistance needs to be compensated by an additional power with the increase in fuel consumption and emission of gaseous pollutants. Furthermore, the maneuvers necessary for the lift, in this adverse condition, and to maintain the desired altitude cause an additional ice accumulation underside of the wings and fuselage. Usually, the de-icing of an aircraft is carried out through a sprayable chemical, in the case of aircraft stopped on the ground, before takeoff or resorting to board mechanisms, specifically studied for the ice removal, which can be classified as mechanical, chemical or thermal. The latter is currently one of the most used. This technology exploits the power of engines conveying hot air from one of the turbine stages to heat the airfoil leading edges [1], resulting in high fuel consumption, and the increase of CO_2_ and NO_X_ in the Earth’s atmosphere. Hence, the current technology is economically expensive, involves a waste of resources and an increase in air pollution. It needs only think, for example, that more than 300,000 tons of CO_2_ are generated daily from the aircraft flights [1]. Thus, there is a growing need for new technologies allowing air decarbonization and low-cost environmentally-friendly flights. Different research approaches have been used to solve the de-icing problem. One of these is inspired by the lotus leaf and the water strider. The passive anti-icing strategy based on hydrophobic surfaces has been investigated, which can retard freezing or frosting and facilitate water droplets sliding off [2,3,4,5]. The contact angle plays a fundamental role, in the hydrophobicity of the surfaces and on the water droplet freezing process. Previous studies [3,6,7,8] have suggested that the freezing start time is delayed due to the smaller solid/liquid contact area, decreasing the heat transfer from the water droplet to the cooling surface. In this context, PDMS polymer, thanks to its low surface energy, seems to turn out to be a good candidate for preparing a hydrophobic surface for anti-icing applications [4,9]. Together with this passive strategy, electro-thermal de-icing methods, at lower power, can be alternative leading technologies to the above-mentioned solution. In this context, for developing a very effective electro-thermal anti/deicing strategy, one of the factors to be taken into account is the possibility to employ the energy source currently already available on the vehicles.

The on-board generators are characterized by a limited power (for example all the Boeing 787 generators produce together 800 KW), and only a part of this energy is reserved to activate the electro-thermal de-icing systems (from 45 to 75 KW) [10]. For instance, according to the reference [10], an heat flux density from 14 KW/m^2^ to 34 KW/m^2^ is needed for a Boeing 787. In recent years, academic and industrial research has increasingly focused on the design of electro-thermal heating systems, which can exploit the advantages of the electrically conductive nanoparticles. In this case, the heating is determined by the Joule effect of the current flowing through the electrically conductive nanomaterial. Joule heating is an energy-efficient method of ice removal that can be capable of real-time de-icing. The choice fell on systems loaded with carbon fillers to replace metal-based electrical heating devices, which present various drawbacks such as high production costs, emission of the electromagnetic wave and oxidative corrosion [11]. Carbon nanotubes [12,13], graphite derivatives [14,15,16,17] and graphene nanoribbons [18] have become the first candidates to develop polymeric matrices or functional coatings able to confer anti/de-icing ability to polymeric systems. Their introduction allowed enhancing the electrical, mechanical and thermal properties [19,20,21]. However, these properties depend on aspect ratio, size, structure and morphology of the nanoparticles dispersed in the composite [19,22]. Many researchers used graphite derivatives or carbon nanotubes to modify the carbon fiber reinforced epoxy composite, improving the electrical conductivity and opening a possibility to use the composite as a heater to de-ice aircraft surfaces. Although the electrical conductivity of the developed nanocharged resins has been found to significantly increase [23,24,25,26] up to value about 10^2^–10^3^ S/m; it should be considered that for these values, the voltage required to promote effective heating, in acceptable times, is still too high compared to the voltage that can be supplied of the currently onboard generators of an aircraft. It is worth noting that for Carbon Fiber Reinforced Composites (CFRCs), which are reinforced with electrically conductive fibers, at least on a theoretical basis, it may be possible to exploit the Joule effect of the current running through the fibers. This strategy is not practicable for Glass Fibers Reinforced Composites (GFRCs), due to the insulating properties of the glass fibers. In this last case, the reinforced epoxy composites must be impregnated with resins containing a high amount of nanofiller, corresponding to a nanofiller concentration beyond the Electrical Percolation Threshold (EPT). This requirement imposes restrictions on the choice of the manufacturing process [27,28] or the control of viscosity, through specific functionalization of the nanofiller, to mitigate the increase in the viscosity due to nanoparticles, hence still making the nanofilled resin processable [29,30,31]. A completely different approach has been recently proposed in literature. It is based on the employment of a film heater used as an interlayer among the layers of GFRCs or CFRCs. In this last direction, the 2D structures, like buckypaper, or graphene-based films, may fulfill requirements such as structural compatibility, flexibility, mechanical reinforcement and adaptability in multilayered structures. Currently, 2D structures can be fabricated by chemical vapor deposition [32], oxide paper-reduction [33,34] and casting [35], to name a few. The graphene/graphite structures are based on stacking processes and orientations of graphitic multilayers allowing high mechanical robustness. This robustness is obtained at the expense of the flexibility of the 2D system. The use of thin thickness allows only apparent flexibility. The introduction of small amounts of an appropriate polymer allows obtaining, at the same time, high mechanical strength and discrete flexibility [36]. In a previous paper [36], it has been proven that the employment of a heating film based on the exfoliated graphite may represent a very efficient strategy for developing anti/de-icing protection systems. For instance, the problem of icing of the aircraft/automotive parts can be efficiently solved using flexible films as a ply of the GFRCs or CFRCs. To prepare this ply, we have tried to optimize all the conditions before dealing with issues related to the scale-up of the technology involving large panels also of curved geometry. In particular, in this work, two types of expanded graphite, commercially available, are used to produce flexible films, by a green solvent casting process, using a PVA-based water solution. The structural investigation has highlighted that the different aspect ratio of two fillers; and the different structure of the starting fillers determine a different heating performance of the obtained films. Higher performance is obtained when the filler with the highest aspect ratio is used. The flexible film heater presents great potentiality as de-icing technology, easily integrable in part of the aeronautical panels or of the aircraft structure.

## 2. Materials and Methods 

**Figure 1:**
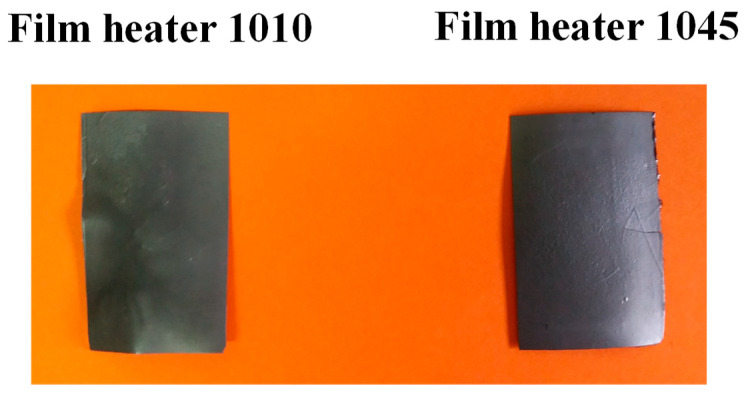
Optical images of the developed film heaters.

Two expanded graphites (supplied by Superior Graphite Co. Chicago, Illinois, USA) have been used for the production of heating films: the ABG 1010 and the ABG 1045. The heater films, based on PVA (Sigma Aldrich—70,000–100,000 MW) (Sigma Aldrich Missouri, USA) have been prepared by a solvent casting process in which the dispersion of filler has been obtained by ultra-sonication (Hielscher model UP200S-24 kHz high power ultrasonic probe) (Hielscher Ultrasonics, Teltow, Germany). Thermal annealing of the film completed the production procedure. In particular, the expanded graphite has been suspended and mixed by magnetic stirring in a solution of deionized water and PVA having a ratio PVA/water (g/mL) of about 0.3 g in 75 mL in order to obtain a good solubilization of the polymer in water. After the solubilization of the polymer, the liquid mixture has been ultra-sonicated for two-hours, allowing to obtain a stable Expanded Graphite (EG) solution. Afterwards, a 200 ± 10 μm-thick flexible film heater has been obtained by evaporation casting, followed by thermal annealing at 120 °C for 1 h applying a pressure of 500 psi. A ratio by weight of expanded graphite/PVA equal to 60/40 has been chosen for both graphites. This specific composition allows obtaining shapeable flexible graphite-based foils. This aspect is relevant because some aircraft parts are more vulnerable than others. Ice accretion during the flight occurs most of all on the leading edge of the aircraft wing and generally covers only 2% of the wing chord [37]. Efficient film flexibility allows its applicability also to localized zones with a curved geometry, such as, for example, the more vulnerable part of an aircraft (leading edges). Films with a ratio by weight of expanded graphite/PVA equal to 70/30 have been also prepared and characterized for both graphites. The results are not reported here because their characterization highlighted that the requirement of flexibility was not satisfied. The film heaters here analyzed, with a ratio by weight of graphite/PVA equal to 60/40 are shown in Figure 1. The expanded graphites and the flexible film heaters have been characterized by different experimental techniques summarized in Table 1. Many of the characterization procedures have been carried out in agreement with those described in a previous paper [36].

## 3. Results and Discussion

### 3.1. Structural Analysis

**Figure 2:**
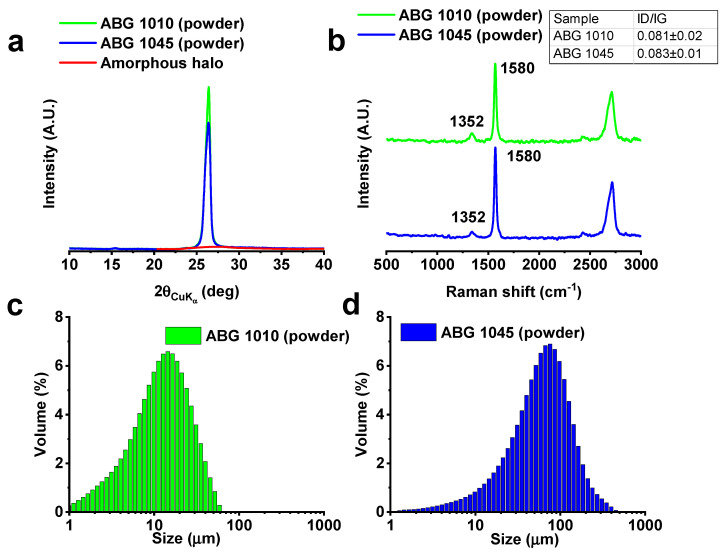
Structural investigation of ABG 1010 and ABG 1045 powders: (**a**) X-ray diffraction, and (**b**) Raman spectra; (**c**) size distribution of ABG 1010, and (**d**) ABG 1045 powders.

X-ray diffractograms of the expanded graphites are shown in Figure 2a. The ratio between the area of the 002 peak (centered at about 2θ = 26°) and of the superimposed amorphous halo (red line, Figure 2a) allowed evaluating the percentage of exfoliated graphite according to a procedure already described in literature [13].

According to the Hermans–Weidinger method [38], the ABG 1010 and ABG 1045 present an exfoliation percentage of about 12% and 14%, respectively. Furthermore, based on Hermans–Weidinger method [38] and on Scherrer’s equation [39], the number of layers for each graphite [40] has been calculated. In particular, considering the value of 3.39 Å for the d-spacing of the reflection (002), a number of about 92 layers for the ABG 1010 and 78 layers for the ABG 1045 has been obtained. The Micro-Raman (MR) spectra of the fillers are shown in Figure 2b. Two intense peaks are detected, one at 1580 cm^−1^, known as “G band” [41] and the other one at 2700 cm^−1^ known as “2D band” [22,42]. Furthermore, the peak related to the “D band” (1352 cm^−1^), associated with the edge distortion phenomena [43] results negligible for both fillers (Figure 2b) due to the presence of graphitic blocks in the filler. The level of disorder in graphene is similar for both expanded graphites. In fact, the average value of the intensity ratio of the D-band (1352 cm^−1^) to G-band (1580 cm^−1^) (ID/IG) is 0.081 ± 0.02 for ABG 1010 system and 0.083 ± 0.01 for ABG 1045 system. The Micro-Raman spectra, not reported here, on the film heaters have shown an additional exfoliation, which may be due to the effect of the ultrasonication process. In fact, higher average values of ID/IG, with respect to the values of fillers, have been obtained. In particular, the values of ID/IG are 0.141 and 0.114 for ABG 1045 and ABG 1010 film heaters, respectively. In order to further define the size of the graphite nanoparticles, an analysis of the frequency distribution has been obtained for both fillers. Different particle sizes, ranging from 1 μm to 450 μm, were observed (Figure 2c,d). In particular, the ABG 1010 is characterized by a size distribution between 1 μm and 60 μm centered at a value of about 14 μm (Figure 2c), whereas the ABG 1045 sample manifests a wider distribution up to values of 450 μm with an average value of 75 μm (Figure 2d). In any case, the ABG 1045 filler, compared with the grade 1010, results to be composed of wider particle sizes, but characterized by thinner thicknesses (see the average number of layers resulting from X-ray analysis). This result, added to the different length perpendicular to the reflection plane 002, observed in the XRD analysis, allows affirming that the two fillers strongly differ in their aspect ratio. Since DLS could overestimate the particle size of the samples, due to the presence of agglomerates, morphological analysis has been carried out to evaluate the size of the graphitic blocks of both expanded graphites.

### 3.2. Morphological Analysis

**Figure 3:**
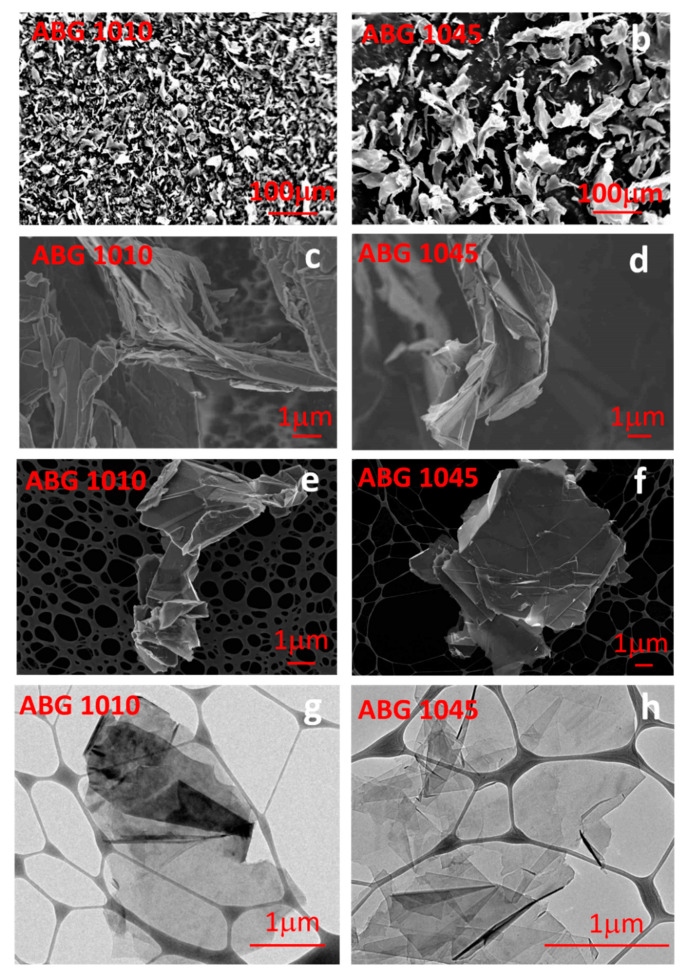
(**a**) SEM micrograph of the exfoliated graphite ABG 1010 raw powder and (**b**) the exfoliated graphite ABG 1045 raw powder; (**c**) magnification of the SEM micrograph of the exfoliated graphite ABG 1010 raw powder and (**d**) magnification of the SEM micrograph of the exfoliated graphite ABG 1045 raw powder; (**e**) FESEM micrograph of the exfoliated graphite ABG 1010 and (**f**) exfoliated graphite ABG 1045 after sonication treatment in isopropanol solution; (**g**) TEM micrograph of the exfoliated graphite ABG 1010, and (**h**) the exfoliated graphite ABG 1045 after sonication treatment in isopropanol solution.

SEM investigation of the expanded graphite ABG 1010 and ABG 1045 samples has been performed to analyze the morphology of each filler before the production of the film heater. Figure 3 shows SEM images of ABG 1010 and ABG 1045 powders, respectively. The expanded graphite nanoparticles appear like a porous structure with graphitic blocks not well separated and folded. The nanosheets of the exfoliated graphite are characterized by highly irregular shapes as already found for this kind of filler [22,36] and due to the thermal treatment of the raw graphite. From a first analysis, the size of ABG 1045 seems to be larger than that of ABG 1010 (Figure 3a,b). The size of the expanded graphite samples was calculated by averaging the size of at least 50 particles using the FESEM images. The images in Figure 3c,d correspond to the graphite samples after a sonication treatment in a 0.1 mg/mL concentration isopropanol solution followed by the drying process at room temperature. Sample ABG 1010 shows a lower size (20.0 ± 11.1 μm) than ABG 1045 filler, which is characterized by an average particle size of 59.7 ± 30.9 μm with a higher standard deviation. These results are in agreement with the analysis of the frequency distribution (DLS). Finally, TEM micrographs (Figure 3e,f) highlight that the ABG 1045 sample result was composed of thinner blocks than the ABG 1010 sample, confirming the X-ray results. In fact, it is well evident, through TEM investigation, that the highest thickness of the filler ABG 1010 hinders the transmission of the electrons, making the TEM image darker. The structural and morphological analysis highlight that the two considered systems differ in their aspect ratio. In particular, from the evaluation of the average size obtained by morphological analysis and the length perpendicular to the reflection 002 obtained by diffractometric analysis, aspect ratio values of about 600 and 2300 have been calculated for ABG 1010 and ABG 1045 filler, respectively. This significant difference has been found relevant in determining the performance of the de-icing phenomenon.

### 3.3. FTIR Spectra Analysis

**Figure 4:**
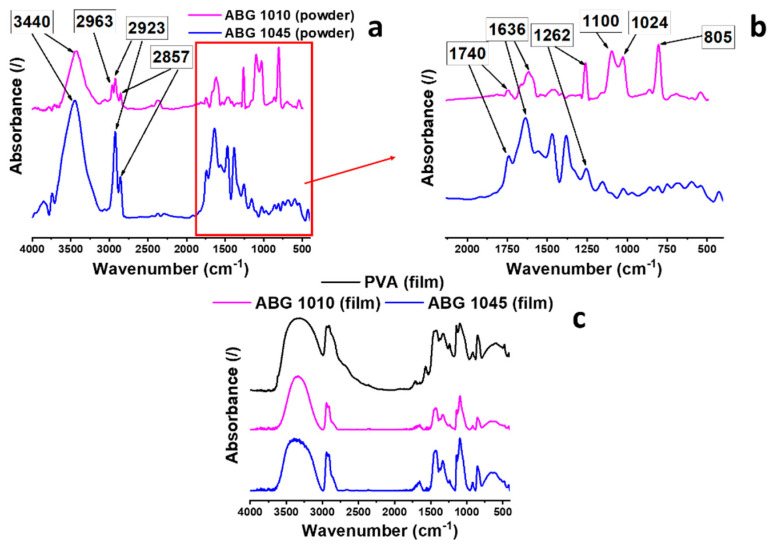
FTIR spectra of the (**a**) fillers; (**b**) magnification of spectra of the fillers in the wavenumber range 2000 cm^−1^–400 cm^−1^; (**c**) film heaters.

Figure 4a shows the FTIR spectra of the two fillers. Both spectra are typical of an oxidized graphite. The broad intense band around 3440 cm^−1^ is due to the O-H stretching vibration. This band is diagnostic of OH groups and/or COOH groups. The weak signals at 2857 and 2923 cm^−1^ in the spectrum of the ABG 1045 powder sample are due to C-H stretching vibrations (asymmetrical and symmetrical stretching of methylene groups, respectively (νasCH_2_ and νsCH_2_). In addition to these signals, the spectrum of the ABG 1010 powder sample also shows the peak at 2963 cm^−1^, indicating the presence of C-H stretching vibrations of methyl groups. The C=C stretching vibration (CC ring) of the graphitic phase is observed at 1636 cm^−1^ [44]. On the left side of this last band, C=O stretching signals are observable (see signals at 1732, 1740 cm^−1^) indicating the presence of carboxyl groups. In the spectral range from 1000 to 1500 cm^−1^, two strong C-O bands at 1100 and 1024 cm^−1^ in the spectrum of the ABG 1010 sample indicate that more oxygenated functional groups are bonded to graphite basal plane of this sample [22]. In addition, the presence of the more intense peak at 1262 cm^−1^ in the spectrum of ABG 1010 sample, also indicates a higher contribution of oxygen due to epoxy rings (all ring bonds stretch and contract in phase). The peak at 805 cm^−1^ is most likely due to the contribution of the same group (asymmetrical ring stretching) [45]. These data highlight that the graphite with higher aspect ratio exhibits a lower amount of oxygen, as expected. In fact, since the oxygen is mostly attached to the edges, the relative content of the edges vs. surface is lower in the graphite with higher aspect ratio. However, for both graphites the presence of oxygenated groups, on the graphitic layers, makes the graphite more hydrophilic, allowing the formation of hydrogen bonds with oxygenated functional groups of the PVA polymer (black line in the range 3000 cm^−1^–3700 cm^−1^ of Figure 4b) [36,44,46].

### 3.4. Physical Analysis of the Film Heaters

**Figure 5:**
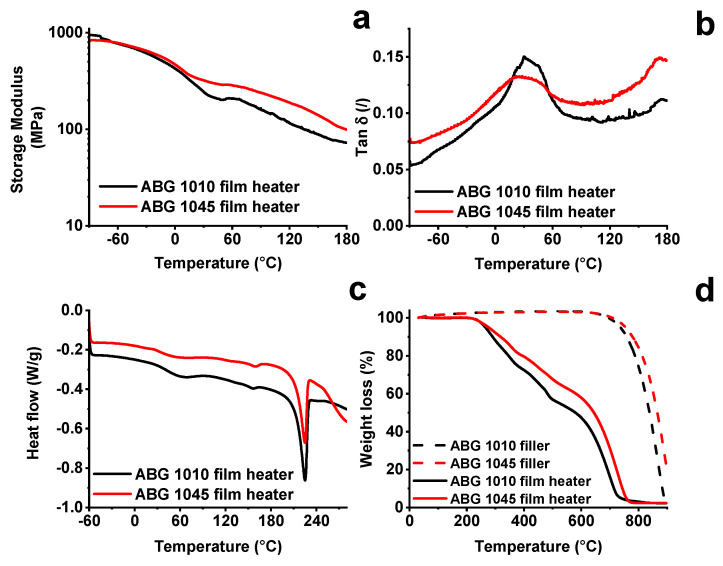
Physical analysis of the film heaters: (**a**) storage modulus as a function of the temperature in the range−60 °C ÷ 180 °C; (**b**) tanδ as a function of the temperature in the range −60 °C ÷ 180 °C; (**c**) Differential Scanning Calorimetry (DSC) curve of the film heaters in the temperature range −60 °C ÷ 300 °C; (**d**) thermogravimetric analysis of the fillers and film heaters.

Dynamic Mechanical Analysis (DMA) has been carried out to characterize the film heaters. The results are shown in Figure 5a,b.

The storage modulus (Figure 5a) progressively decreases up to 180 °C. The change in shape of the curve profile in the range between 20 °C and 40 °C is related to glass transition temperature (T_g_) of the polymeric component of the film. A glass transition temperature (T_g_) value of ~30 °C is confirmed by the presence of a peak in the profile of tanδ (Figure 5b). Although the PVA is characterized by a low glass transition temperature, both film heaters manifest higher storage modulua, even above 0 °C. Differential Scanning Calorimetry (DSC) and Thermogravimetric Analyses have also been carried out for the characterization of the thermal properties of the film heaters. The results are shown in Figure 5c shows that the PVA component of the films melts in the temperature range between 200 °C and 235 °C, where an endothermic peak centered around 224 °C is observed. Figure 5d highlights that, as expected, the film heaters degrade in a range of temperatures lower than the respective fillers. In particular, ABG 1045 filler is characterized by greater thermal stability compared to the ABG 1010 filler, which can be quantified in about 30 °C as shown by the shift of the mid-point (50% weight-loss temperature) from 840 °C, for the ABG 1010 sample, to 870 °C for ABG 1045. The higher stability of ABG 1045 is also obtained for the film systems, where an increase in the midpoint of about 70 °C is observed. The onset-degradation temperature (5% weight-loss temperature) shifts from a value of 265 °C for the ABG 1010 based system to a temperature value of 280 °C for the ABG 1045 based system, with a gain in terms of thermal stability of about 15 °C. These results perfectly agree with the FTIR results of Figure 4a, which highlights a higher concentration of oxygenating groups on the graphite ABG 1010 characterized by a lower aspect ratio. In any case, both the heating films are thermally stable up to 200 °C. The electrical conductivity of graphene film heaters was measured using the four-wire technique. The values 3.0 × 10^3^ S/m and 4.7 × 10^2^ S/m were obtained for ABG 1045 and ABG 1010 based systems, respectively. Although the two fillers appear almost similar in many aspects, the film heaters based on the different graphites differ in electrical conductivity by an order of magnitude. This is probably due to the different aspect ratio of the two fillers. In fact, the ABG 1045 filler is characterized by a greater degree of expansion with respect to ABG 1010 filler. This allows the formation of more efficient percolation pathways, which determine a greater electrical conductivity. In a previous work [22], a little (4% wt/wt) variation in the exfoliation degree of a 2D filler led to a difference in electrical conductivity of about 12 orders of magnitude, for an amount of filler near the Electric Percolation Threshold (EPT) of the system. In this last case, the difference in electrical conductivity can be ascribed to an increase in the expansion degree of the starting filler. The difference in electrical conductivity is lower compared to the previous referenced case, because in the film heaters here discussed, the amount of the filler is 60% by weight, therefore a filler concentration far beyond the EPT. Debelak et al. [47] highlighted that an increase in the granulometry and size of the graphite particles allows a reduction in the electrical resistance in an epoxy resin-based system. Hence, the factor that influences the electrical conductivity of the two systems is most likely due to the characteristic sizes of the graphitic blocks. The ABG 1045 filler is characterized by a size of the graphitic blocks larger than the ABG 1010 filler, as also evidenced by the different values of the aspect ratio.

### 3.5. Electrical Heating Behavior (Constant Current)

**Figure 6:**
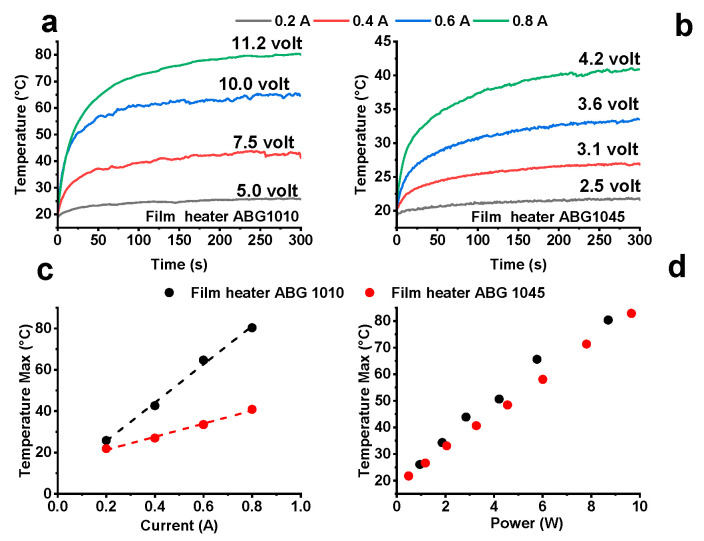
Temperature–time plots at different applied current for (**a**) the ABG 1010 film heater; (**b**) the ABG 1045 film heater; (**c**) T_max_ vs. applied current of the film heaters at room temperature; (**d**) T_max_ vs. applied power of the film heaters at room temperature.

The electrical heating behavior of the two considered films was investigated by applying different constant current from 0.2 to 0.8 A. Two Cu foils strips, acting as electrodes, with a thickness of 80 mm were adhered on both sides of film/paper, in order to apply a suitable electric current density and hence evaluate the heating temperature by the use of thermocouples appositely placed on both surfaces of the film heater. Figure 6a,b show the time dependent-temperature profiles of the two systems. The ABG 1010 film heater, for the same current value, allows faster heating and higher values of the maximum temperature than the ABG 1045 system. The maximum temperatures (T_max_) of the two films, at different constant currents, are shown in Figure 6c. The T_max_ values increase linearly with the applied current, but with different slopes. This different detected slope can lead to incorrect assessment of the effectiveness of the two systems. For a proper evaluation on the comparison between the different systems, the relationship between the applied electric power and maximum temperature values should be considered. The electric power, defined by the product of the voltage (V) and the current (I), P = VI, is converted into heat by the Joule heating process. Figure 6d shows a linear relationship between the electric power and the maximum temperature obtained, in the electric power range from 0.5 W to 10 W. For the same value of power, the graph highlights almost the same temperature values for both systems. In light of the above considerations, the two systems can be considered equivalent and in both cases, a desired steady-state maximum temperature can be controlled by adjusting the applied electric power.

**Figure 7:**
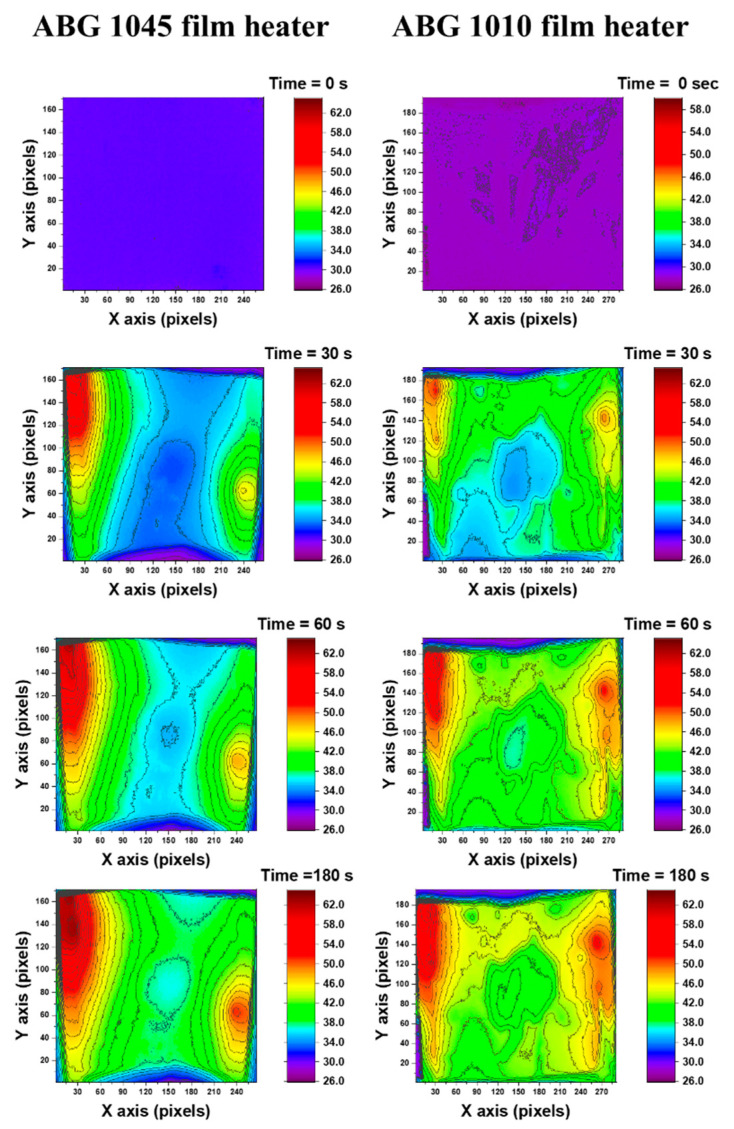
Thermal images of the two film surfaces at different times (initial step, after 30 s, 60 s and 180 s).

**Figure 8:**
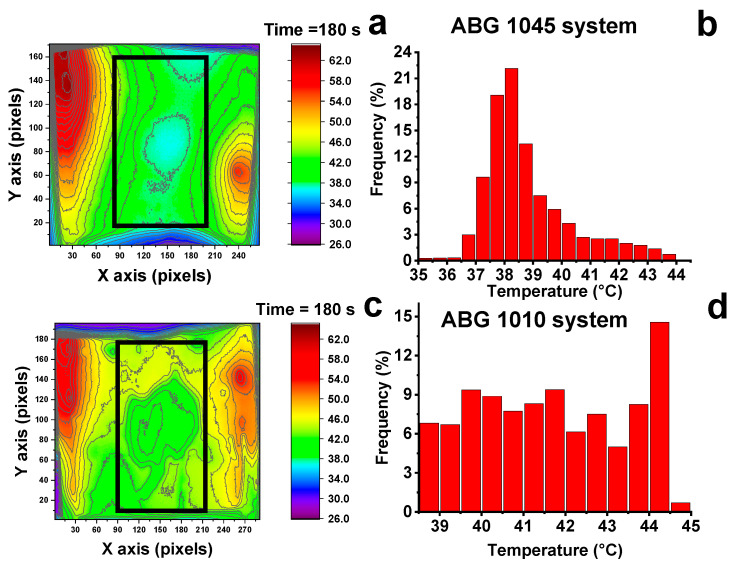
(**a**) Thermal images on the film surface of the ABG 1045 film heater; (**b**) surface temperature distribution of the ABG 1045 film heater; (**c**) Thermal images on the film surface of the ABG 1010 film heater; (**d**) surface temperature distribution of the ABG 1010 film heater.

To better understand the heat generation and heat transfer process, an infrared camera was used to observe the performance of both systems. In particular, in Figure 7, the temperature mapping of the film heaters (dimensions 0.2 × 7.5 × 5 cm^3^) vs. time during heating has been monitored applying the same constant power of 2 W. The samples were positioned vertically, keeping the two film faces isolated from the contact state with other corps, allowing to have the same boundary conditions on both faces of the film exposed to the air. The pictures were taken at different times in order to evaluate the evolution of the heating process on the surfaces of the films. In particular, the investigation has been performed in the commencement of heating (initial step, and after 30 and 60 s) and in the steady-state condition, which is reached after 180 s of heating. In both systems, the hottest zones are those where the copper contacts are located. The greater electrical conductivity of the copper contact, with respect to the heater film, determines a more effective local heating. In this way, a temperature gradient is created between the edges zones (next to the copper contacts) of the sample and the central part. For this reason, a further heating phenomenon is obtained besides that obtained by the Joule effect. The images of Figure 7 show that the ABG 1045 based system is characterized by a surface temperature distribution more homogeneous compared with the ABG 1010 system. The uniformity is visible in the central area of the film, which acquires the same color tone at a longer time. The ABG 1010 based sample, unlike the ABG 1045 film heater, presents discontinuities in the color also in the central zone of the sample even after 180 s. In order to highlight this aspect, an analysis of the average temperature in the central area of the two samples analyzed was carried out. Figure 8 shows the thermal image of the ABG 1010 and ABG 1045 systems, after 180 s while an electrical power of 2 W is applied.

The distribution of the temperature values, detected considering the pixels in the region delimited by the black square line, is shown in the Figure 8b,d. Although the average temperature values are quite similar (about 38 °C for the ABG 1045 system and about 41 °C for the ABG 1010 system), the two systems differ in the temperature distribution value. In fact, the ABG 1045 system presents a narrower distribution temperature and the central region of the film (Figure 8a) with a more homogeneous color tone with respect to the ABG 1010 system (see Figure 8c). Bearing in mind that the energy supplied to the two systems is the same (2 W) and that the dimensions of the samples are similar, the different heating conduction along the surface probably is due to the different interconnection between the adjacent graphitic sheets, in the PVA matrix. This interconnection is a function of the structure of the fillers, which present different size and aspect ratio, as described above in the morphological and structural analysis. 

### 3.6. Electrical Heating Behavior (Constant Voltage)

**Figure 9:**
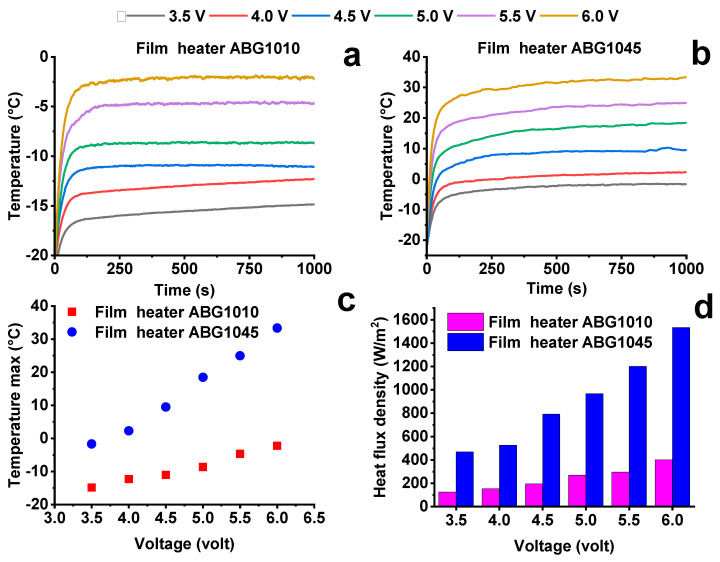
Temperature–time plots at −20 °C at different voltage for (**a**) the ABG 1010 film heater; (**b**) the ABG 1045 film heater; (**c**) T_max_ vs. applied voltage of the film heaters at −20 °C; (**d**) T_max_ vs. applied power of the film heaters at −20 °C.

The last electrical heating analysis was carried out by applying different constant voltages from 3.5 V to 6.0 V, at the environmental temperature of −20 °C. This investigation allows evaluating the applicability of the heater systems, considering the capacity of the generators on-board the aircraft and the large areas affected by the icing phenomenon. The onboard generators supply an alternating voltage of 115/230 V (similar to that of a domestic electrical appliance) while the onboard control units require a direct voltage of 28 V (comparable to that available in a car). A converter reduces the alternating voltage of the 115/230-volt generators to achieve an alternating voltage of 28 V, and a transformer/rectifier then rectifies that to a 28 V direct voltage [48,49,50]. Figure 9a,b show the trend of the temperature versus time of the film heaters ABG 1010 and ABG 1045, respectively. 

The ABG 1045 based system reaches maximum temperatures higher than those of the ABG 1010 based system (Figure 9c) for the same voltage value. The latter system, in the considered range voltage, does not reach the temperatures required for de-icing (T > 0 °C). On the contrary, the ABG 1045 based system, having a higher electrical conductivity, allows for the same applied voltage (Figure 9c) to supply sufficient power to achieve de-icing. The heat flux density detected for ABG 1045 film heater, for all the voltage values able to give de-icing, and displayed in Figure 9d, is lower than those required for example for a Boeing 787, which needs values of heat flux densities from 14 KW/m^2^ to 34 KW/m^2^) [10]. On the bases of this consideration, a comparison of the energy consumption between a classical heating de-icing technology and the developed film heater can be performed. In fact, a silicone rubber wire heater [51] needs 23250 W/m^2^ to raise the surface temperature from −0.5 °C to 34.4 °C in 90 s. The use of a rubber wire heater to heat an area equal to that of the samples obtained in this study, would need about 94 W. The use of 6 V with the ABG 1045 system would allow reaching, in about 90 s, the temperature of 25 °C, starting from an environment temperature significantly lower than 0 °C (−20 °C) with a power equal to 6 W. Film heaters similar to those here described are developed in the literature, but pay little attention to technological parameters such as the suitable voltage on-board the aircraft and/or car. Yan et al. [52] proposed a composed bilayer film of multi wall carbon nanotubes (MWCNT) and polydimethylsiloxane which can reach temperatures up to 200 °C considering voltage values up to 100 V. In particular, temperatures similar to those obtained in this work (80 °C), in the same boundary conditions (room temperature) were obtained with a voltage of about 60–70 V. In another study, resins loaded with graphene nanoplatelets have been proposed as self-heating coatings of composite materials [53]. The low concentrations of conductive used fillers allow good de-icing performance only with applied voltages of about 700–800 V. In this work, the detected performance highlights that the obtained film heater can be a valid alternative to known resistance heating pad thermal systems as silicone rubber wire heaters and different other film heaters proposed in literature. Further de-icing tests were carried out at an environmental condition of about −22 °C and at a voltage range from 8 V to 11 V in order to evaluate the de-icing time of a predefined volume of ice.

**Figure 10:**
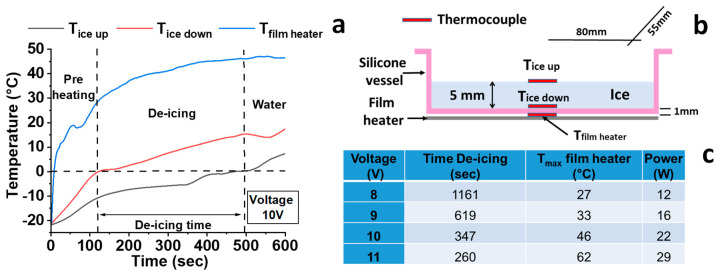
(**a**) Temperature behavior vs. time during de-icing test; (**b**) schematic configuration of the de-icing test; (**c**) parameter of the de-icing test.

The de-icing test configuration is shown in Figure 10b, in which the ABG 1045 film heater has been positioned below 1 mm thick silicone vessel, filled with a 5 mm thick ice volume. The placement of three thermocouples positioned as in the scheme of Figure 10b allows evaluating the evolution of the ice and of the heater film temperature versus time (see Figure 10a). At the beginning, the temperature linearly increases rapidly. While the temperature of the film continues to increase, the trend of the temperatures detected above and below the volume of ice shows a change in slope near 0 °C. This phenomenon is due to the ice phase change, which melts into water. The phase change lasts a few seconds, during which the temperature remains constant at 0 °C due to the ice latent fusion heat. After this period, the temperature starts to increase again, a sign that the ice in direct contact with the thermocouples has melted. The difference between the spent time in order that the thermocouple placed above the ice volume detects the temperature of 0 °C and the spent time in order that the thermocouple positioned below the ice volume detects the same temperature, is de-icing time. An increase in the voltage value allows an increase in the applied power and a consequent reduction in the freezing times, as shown in Figure 10c. The poor thermal conductivity of the silicone vessel creates a thermal gradient of about 30–35 °C between the temperature of the ice and that of the heater. The high-temperature resistance of the film heater, up to 180 °C, (see “Physical analysis of the film heaters” section) can allow the thermal gap far higher.

## 4. Conclusions

In this work, the effect of the size of expanded graphite particles on the electric heating and de-icing performance of flexible films based on polyvinyl alcohol has been studied. An increase in the aspect ratio of the filler allows obtaining an increase in the de-icing performance of the heater film. It generates higher temperature values, using lower applied voltages. This result is crucial when considering the applicability of the heating system in several sectors, like the aeronautical and automotive fields, in which the requirements of the generators’ capacities on-board are predetermined. The de-icing tests have shown that the de-icing times are relatively low using low applied voltage values and the temperatures detected on the film are in the thermal stability range of the composite film. The de-icing technology here proposed allows saving energy and resources making the current generators already applicable for de-icing operations.

## Figures and Tables

**Table 1:** Characterization methods.

Method	Device	Characterized Sample	Procedure Measurement/ Technical Specifications
Wide-angle X-ray diffraction (WAXD)	Bruker D8 Advance diffractometer (Bruker, Billerica, MA, USA)	Fillers	According to ref [36]
Scanning Electron Microscopy (SEM)	JSM-6700F, (JEOL Akishima, Tokyo, Japan)	Fillers	According to ref [36]
Transmission Electron Microscopy (TEM)	JEOL model JEM-1400 Plus (JEOL Akishima, Tokyo, Japan)	Fillers	Resolution image 0.38 nm between dots and 0.2 nm between lines.
Raman Spectroscopy	Renishaw inVia (Renishaw Wotton-under-Edge, U.K.)	Fillers	According to ref [36]
FTIR spectroscopy	BRUKER Vertex70 (Bruker, Billerica, Ma, USA)	Fillers/film	According to ref [36]
Dynamic mechanical analysis (DMA)	Tritec 2000 DMA-Triton Technology (Triton Technology, Grantham, UK)	Film (0.3 × 10 × 15 mm^3^)	Three points bending mode Amplitude = 0.05 mm; According to ref [36]
Differential Scanning Calorimetry (DSC)	Mettler DSC 822/400 (Mettler Toledo) (Mettler-Toledo Columbus, OH, USA)	Fillers/film	According to ref [36]
Thermogravimetric analysis (TGA)	Mettler TGA/SDTA 851 (Mettler-Toledo Columbus, OH, USA)	Fillers/film	According to ref [36]
Dynamic Light Scattering (DLS)	Zetasizer ZSP (Malvern Panalytical Ltd. Malvern, U.K.)	Fillers	Refraction index (1.33–2.4)
Electrical conductivity measurement	HP 34401A Digital Multimeter (Keysight Technologies, Santa Rosa, Ca, USA)	Film (0.2 × 7.5 × 1 cm^3^)	4-wire method According to ref [36]
Temperature measurement	-Thermocouples (Omega Engineering Ltd. Manchester U.K.) -Power supply (EA-PS 2042-20B) (EA Elektro-Automatik GmbH and Co.KG Helmholtzstr, Viersen)	Film (0.2 × 7.5 × 5 cm^3^)	According to ref [36]
Temperature distribution monitoring	Imager camera PCE-PI 450 (PCE Deutschland GmbH Meschede, Germany)	Film (0.2 × 7.5 × 5 cm^3^)	Frequency = 27 Hz IR resolution of 382 × 288 pixels
